# Influence of positive and negative affect on self-management among patients with early chronic kidney disease during the COVID-19 pandemic: The mediating and suppressing effect of ego depletion

**DOI:** 10.3389/fpsyt.2022.992404

**Published:** 2022-09-29

**Authors:** Yi Cui, Rong Li, Tianqi Yang, Hua Wang, Shasha Jin, Na Liu, Hongbao Liu, Yinling Zhang

**Affiliations:** ^1^Department of Nursing, Air Force Medical University, Xi'an, China; ^2^Department of Nephrology, The First Affiliated Hospital of Air Force Medical University, Xi'an, China; ^3^Department of Military Medical Psychology, Air Force Medical University, Xi'an, China; ^4^Department of Nephrology, The Second Affiliated Hospital of Air Force Medical University, Xi'an, China

**Keywords:** chronic kidney disease, ego depletion, positive and negative affect, self-management, COVID-19, mediating effect, suppressing effect

## Abstract

**Background:**

Self-management in patients with early chronic kidney disease (CKD) can effectively delay damage to renal function. However, with the continuous spread of COVID-19, patients cannot receive timely treatment, which can lead to different affects, resulting in ego depletion and serious challenges to self-management. This study aimed to investigate the mediating and suppressing roles of ego depletion on the relationship between positive and negative affect and self-management among patients with early CKD during the COVID-19 pandemic in China.

**Methods:**

A total of 383 patients with early CKD from three tertiary hospitals were enrolled by convenience sampling in our cross-sectional study from September 2021 to March 2022. Participants completed the Sociodemographic Questionnaire, Positive Affect and Negative Affect Scale, Self-Regulating Fatigue Scale and Chronic Kidney Disease Self-Management Instrument. A structural equation model was conducted to test the mediating and suppressing effects of ego depletion on the relationship between positive and negative affect and self-management.

**Results:**

The average score of the participants' self-management was 84.54 (SD: 19.72), and nearly 60% of them were at low and moderate levels. The mediating effect of positive affect on self-management through ego depletion was significant (β = 0.248, 95% CI: 0.170 to 0.376), accounting for 53.22% of the total effect. The suppressing effect of negative affect on self-management through ego depletion was significant (β = −0.191, 95% CI: −0.310 to −0.118), and the absolute value of the ratio of the suppressing effect to the direct effect was 66.55%.

**Conclusions:**

Ego depletion partially mediated the relationship between positive affect and self-management while suppressing the relationship between negative affect and self-management among patients with early CKD during the COVID-19 pandemic. The reduction of patients' ego depletion must be taken as the intervention target to improve self-management and delay the progression of CKD.

## Introduction

Chronic kidney disease (CKD) is a lifelong disease and is defined as renal structural or functional abnormalities or unexplained decreases in glomerular filtration rate (< 60 ml/min/1.73 m^2^) for more than 3 months. It is categorized into five stages based on the estimated glomerular filtration rate (eGFR) (1). Generally, early-stage CKD encompasses stages 1 to 3a (2). CKD has become a world public health problem seriously endangering human health and is another major hazard to the physical and psychological health of patients after cardiovascular disease, diabetes and malignant tumors (3). According to data from the World Health Organization, the global mortality rate of CKD is expected to increase by approximately 14% by 2030 (4). It has been reported that the global prevalence rate of CKD is 14.3% (5), and the prevalence rate in China is 10.8% (6). The number of patients with CKD in the world has reached 697.5 million, and patients with stage 1–3 CKD accounted for 97.8% of all patients by 2017. Among them, China had 132.3 million patients, making it the country with the largest number of patients with CKD worldwide (7). Due to the long course of CKD, the vast majority of patients need home treatment and rely on themselves to solve physical and psychological problems. Studies have shown that timely treatment and effective self-management in the early stage of CKD can significantly delay further damage to renal function (8). However, since the subjective symptoms of patients with early CKD are not obvious, patients do not pay enough attention to the disease. In addition, during the coronavirus disease 2019 (COVID-19) pandemic, the increased risk of disease infection, road closures and stay-at-home orders limited the physical activity of patients with CKD, resulting in patients not being able to go to the hospital for checkup and treatment in time; the restriction of food supplies during lockdown also changed the patients' previous good dietary habits; continued lockdown has the potential to increase difficulty for patients seeking to refill prescriptions as well, thereby undermining their self-management (9). Improving self-management has become an urgent priority for patients with early CKD.

Self-management means that patients with early CKD, under the guidance of medical staff, must acquire the skills and confidence to deal with the disease through learning, actively participating in health care activities, and maintaining good physical and psychological health (10). Self-management can significantly delay the course of disease, reduce the incidence of complications, and effectively prevent progression from the early stage to the end stage (11). In the long-term self-management of CKD, a stable condition of the disease and support from family and friends can produce positive affect. In contrast, recurrent attacks of the disease, limited physical activity and expensive treatment can lead to negative affect such as fear, anxiety, and depression (12). Especially during the ongoing outbreak of COVID-19, patients' conditions cannot be monitored, treatment cannot be adjusted, and medicines cannot be supplemented in a timely manner, which is likely to cause helplessness and even panic (13, 14). Studies have shown that both positive and negative affect are correlated with patients' self-management. Travado (15) found a significant positive correlation between positive affect and self-management. Song (16) found that negative affect was negatively correlated with self-management. However, some studies have proposed that negative affect can activate personal self-management behavior (17). As a result, there are still different views on the relationship between negative affect and self-management.

The theory of ego depletion, also known as self-regulating fatigue, refers to a state of limited and internal consumption of psychological resources caused by individual self-regulating behaviors, resulting in a series of cognitive, emotional, and behavioral problems (18). Its core mechanism includes the following aspects: individuals need to consume resources to carry out activities; such resources are limited; and the success of activities is affected by the amount of such resources. The more abundant the resources are, the more likely activities will be carried out successfully; the process of performing an activity is a process of temporarily expending resources, which can be restored after a proper rest; and different activities require the same kind of resources, and if resources are consumed in one activity, then the actual resources available for another activity will be reduced (19, 20). According to this theory, the expression of positive and negative affect in self-management, the decision-making about treatment plans and the management of self-behavior of patients with early CKD consume their psychological resources and energy, resulting in ego depletion. Especially in the face of a major epidemic, patients' ego depletion is more serious. Previous studies have confirmed that patients with chronic diseases show high ego depletion and have a more profound and lasting impact (21). When individuals' cognitive, decision-making and behavioral control abilities decrease, they are more inclined to express negative emotions and are prone to poor living habits and impulsive behavior, and their self-management is reduced (22). Shan (23) found that there was a negative correlation between positive affect and ego depletion. Xiong (24) found that people with high ego depletion showed a more obvious attention bias toward negative emotional pictures than people with low ego depletion. Moreover, Hsu (25) found that the lower the ego depletion, the better the self-management of patients with heart failure. It was also found that hematological patients with severe ego depletion had lower self-management and were prone to escape coping strategies (26). Thus, ego depletion may play an indispensable role in the relationship between positive and negative affect and self-management.

Previous studies have shown that positive and negative affect, ego depletion and self-management are correlated with each other. However, the mechanism of positive and negative affect on self-management has not been discussed among patients with early CKD in China, especially during the continuous period of the COVID-19 pandemic. Therefore, the main purpose of this study was to explore the mechanism of positive and negative affect on self-management and investigate the mediating or suppressing role of ego depletion on the relationship between them. There are four hypotheses in our study: (H1) Positive affect of patients with early CKD in China is positively related to their self-management; (H2) Negative affect of patients with early CKD is positively related to their self-management; (H3) Ego depletion plays a mediating role in the relationship between positive affect and self-management; and (H4) Ego depletion plays a suppressing role in the relationship between negative affect and self-management.

## Materials and methods

### Participants and procedures

This was a cross-sectional and descriptive study conducted among patients with early CKD from three tertiary hospitals in China, by the convenience sampling method from September 2021 to March 2022. The inclusion criteria were as follows: (1) age ≥18 years; (2) diagnosis of stage 1-3a CKD according to the National Kidney Foundation-Kidney Disease Outcomes Quality Initiative (K/DOQI) guidelines; (3) clear consciousness and normal communication skills; and (4) informed and voluntary participation in this study. The exclusion criteria were patients who (1) had cognitive impairment or complications with mental illness; (2) had serious cardiovascular, nervous, respiratory and other system diseases; and (3) were unable to complete the questionnaire. The sample size should be 5~10 times that of the independent variables, which was calculated based on the principle of Kendall estimation (27). There were 37 independent variables in this study. Considering a 20% sample loss rate, the minimum sample size was [37 × 5 × (1+20%)] = 222. On the other hand, Mueller (28) proposed that the sample size of the structural equation model should be more than 200. Finally, a total of 383 patients with early CKD were recruited for the study.

Participants were recruited from the Department of Nephrology after approval by the Ethics Committee of the Second Affiliated Hospital of Air Force Medical University (No. 202206-02). Data were collected in face-to-face interviews by researchers. First, the researchers used unified instructions to explain the purpose and significance of this study to the patients who met the inclusion criteria and asked for their written informed consent. Then, the participants were invited to complete the structured questionnaires by themselves in the ward. It took them 15–20 min to finish the questionnaires. The questionnaires were checked by researchers carefully once they were submitted to ensure that there was nothing missing or wrong. During the investigation, participants could withdraw at any time.

### Measures

#### Sociodemographic questionnaire

The questionnaire was designed by the researchers in line with the purpose of the study and consisted of items on age, gender, marital status, education level, residence, resident manner, provider payments, monthly income in Chinese yuan, course of disease, review of the frequency, number of hospitalizations for CKD, stage of CKD, and BMI index.

#### Positive affect and negative affect scale

The Positive Affect and Negative Affect Scale was used to assess the participants' emotional experience in the past 1–2 weeks. The scale was designed by Watson (29) and revised in Chinese by Qiu (30) to include 18 positive and negative affect items. Each item was measured on a 5-point Likert scale, ranging from 1 (“never”) to 5 (“almost all the time”). In this study, Cronbach's α = 0.917 and 0.840 for the positive affect subscale and negative affect subscale, respectively.

#### Self-regulating fatigue scale

The Self-Regulating Fatigue Scale was developed by Australian scholar Nes (31) and adapted to Chinese by Wang (32). The scale was used to reflect an individual's specific depletion, including 16 items in three dimensions (cognition, emotion, and behavior). Each item was measured on a 5-point Likert scale, with a score of 1–5 from “strongly disagree” to “strongly agree.” The higher the score was, the more serious the individual's ego depletion. The Cronbach's α of the scale in this study was 0.788.

#### Chronic kidney disease self-management instrument

The Chronic Kidney Disease Self-Management Instrument (CKD-SM) is a scale for the self-management behavior of patients in the early stage of CKD (stage 1–3). The scale was developed by Lin (33) and revised in Chinese by Liu (34) to include four dimensions of self-integration, problem solving, seeking social support and adherence to the recommended regimen with 29 items. Each item was measured on a 4-point Likert scoring system. The higher the score was, the better the participants' self-management behavior. The scale had good stability and high reliability and has been recognized internationally. In this study, the Cronbach's α of the scale was 0.955.

### Statistical analysis

The data were analyzed by SPSS for Windows Version 26.0. Descriptive statistics were conducted to describe sociodemographic characteristics, positive and negative affect, ego depletion and self-management. Self-management, ego depletion, positive affect and negative affect are all continuous variables. The normality of all variables in our study was examined by the Kolmogorov-Smirnov (K-S) test and skewness and kurtosis tests. All the data were normally distributed. We performed the comparison of self-management among different sociodemographic subgroups using independent *t-test* or one-way ANOVA. In addition, we explored the correlation among study variables by using Pearson's correlation coefficient (*r*). We set the statistical significance level at 0.05 for all analyses (*p* < 0.05).

Mplus Version 8.3 was used to establish the structured equation model. Positive and negative affect were independent variables, ego depletion was a mediating variable, and self-management was a dependent variable. The maximum likelihood method was used to evaluate the measurement model, and the bootstrap 95% confidence interval (95% CI) was used to test whether the regression coefficients were significant for estimating the mediation effect from 1,000 samples (35). If the 95% CI of the indirect effect did not contain zero, then the indirect effect was considered significant.

## Results

### Common method deviation test

We performed the Harman Univariate Test to test the common method deviation. All variables were put into an exploratory factor analysis to determine the fewest factors necessary to explain the variation in variables. If only one factor was separated out or the explanatory power of a factor was particularly large, we assumed a serious common method deviation. In our study, there were 11 factors with eigenvalues > 1. The first factor explained 27.472% of the total variance, which was lower than the critical value of 40%. Therefore, there was no common method deviation in the data used in our study.

### Sociodemographic characteristics and the comparison of self-management among the participants

The results of the sociodemographic characteristics and the comparison of self-management among the participants are shown in Table 1. A total of 383 patients with early CKD were involved in our study, with an average age of 45.93 ± 16.07 years (range = 18–89). More than half of the participants were male (60.31%, *n* = 231). The majority of participants were married (77.03%, *n* = 295). A total of 56.92% of the participants were at stage 1 of CKD, and 60.31% had been diagnosed for more than 12 months. The mean scores for self-management, ego depletion, positive affect and negative affect were 84.54 (SD: 19.72), 43.66 (SD: 8.07), 22.61 (SD: 7.07), and 18.55 (SD: 4.91), respectively. For self-management, nearly 60% of patients with early CKD were at low and moderate levels.

**Table 1:** Sociodemographic characteristics of the participants and the comparison of self-management behavior (*n* = 383).

**Variables**	***n* (%)**	**Mean**	**SD**	***t/F***	***p***
**Age**				*F* = 22.364	< 0.001**
18~45	190 (49.61%)	90.98	16.81		
46~69	163 (42.56%)	78.16	20.24		
70~	30 (7.83%)	78.43	21.31		
**Gender**				*t* = −1.696	0.091
Male	231 (60.31%)	83.16	19.93		
Female	152 (39.69%)	86.64	19.28		
**Marital status**				*F* = 1.974	0.117
Unmarried	67 (17.49%)	89.84	17.54		
Married	295 (77.03%)	83.38	20.29		
Divorced	11 (2.87%)	83.55	17.91		
Widowed	10 (2.61%)	84.40	13.92		
**Education level**				*F* = 14.356	< 0.001**
Junior high school or less	188 (49.09%)	80.69	20.18		
High school degree	123 (31.11%)	84.40	19.29		
Undergraduate or above	72 (18.80%)	94.85	15.27		
**Residence**				*t* = 2.848	0.005**
Urban	220 (57.44%)	86.99	19.13		
Rural	163 (42.56%)	81.24	20.08		
**Resident manner**				*t*=-1.862	0.066
Live with family	329 (85.90%)	83.91	20.26		
Live alone	54 (14.10%)	88.39	15.65		
**Provider payments**				*t* = −3.115	0.003**
Medical insurance	347 (90.60%)	83.81	20.12		
Self-paying	36 (9.40%)	91.64	13.62		
**Monthly income (CNY)**				*F* = 5.428	0.005**
< 2,000	110 (28.72%)	81.43	19.31		
2,000~5,000	198 (51.70%)	83.89	19.79		
>5,000	75 (19.58%)	90.84	18.98		
**Course of disease**				*t* = 3.227	0.001**
3~12 months	152 (39.69%)	88.34	16.92		
>12 months	231 (60.31%)	82.05	21.03		
**Frequency of the review**				*F* = 10.181	**<** 0.001**
< 1 month	116 (30.29%)	88.88	16.80		
1~3 months	122 (31.85%)	87.10	18.33		
>3 months	145 (37.86%)	78.92	21.71		
**Number of hospitalizations**				*F* = 3.090	0.047*
0	173 (45.17%)	86.12	19.52		
1~2	133 (34.73%)	85.35	18.96		
≥3	77 (20.10%)	79.62	20.90		
**Stage of CKD**				*F* = 0.343	0.710
1	218 (56.92%)	85.13	20.28		
2	93 (24.28%)	83.11	20.29		
3a	72 (18.80%)	84.61	17.28		
**BMI index**				*F* = 1.070	0.362
< 18.5	17 (4.44%)	86.24	21.79		
18.5~23.9	163 (42.56%)	86.31	20.06		
24~27.9	140 (36.55%)	82.32	18.46		
≥28	63 (16.45%)	84.46	20.93		

CKD, chronic kidney disease; CNY, Chinese Yuan; BMI, body mass index; SD, standard deviation. * Statistically significant value of *p* (< 0.05); ** Statistically significant value of *p* (< 0.01).

For self-management among patients with early CKD, we found that there were significant differences in age (*F* = 22.364, *p* < 0.001), education level (*F* = 14.356, *p* < 0.001), residence (*t* = 2.848, *p* = 0.005), provider payments (*t* = −3.115, *p* = 0.003), monthly income in Chinese Yuan (*F* = 5.428, *p* = 0.005), course of disease (*t* = 3.227, *p* = 0.001), frequency of review (*F* = 10.181, *p* < 0.001), and number of hospitalizations (*F* = 3.090, *p* = 0.047) but found no significant differences in other sociodemographic characteristics.

### Correlation analysis of positive affect, negative affect, ego depletion and self-management

The Pearson correlation analysis of all variables is presented in Table 2. The results showed that positive affect was significantly negatively correlated with ego depletion (*r* = −0.442, *p* < 0.01) and positively correlated with self-management (*r* = 0.455, *p* < 0.01). Negative affect was significantly positively correlated with ego depletion (*r* = 0.270, *p* < 0.01) and self-management (*r* = 0.140, *p* < 0.01). Ego depletion was significantly negatively correlated with self-management (*r* = −0.438, *p* < 0.01).

**Table 2:** Pearson correlation analysis of self-management, ego depletion, and positive and negative affect (*n* = 383).

	**M**	**SD**	**1**	**2**	**3**	**4**	**5**	**6**	**7**	**8**	**9**	**10**	**11**
1. Self-management	84.54	19.72	—	—	—	—	—	—	—	—	—	—	—
2. Self-integration	33.97	8.13	0.933**	—	—	—	—	—	—	—	—	—	—
3. Problem solving	25.74	7.71	0.943**	0.841**	—	—	—	—	—	—	—	—	—
4. Seeking social support	12.74	4.31	0.826**	0.676**	0.768**	—	—	—	—	—	—	—	—
5. Adherence to recommended	12.10	3.28	0.400**	0.263**	0.223**	0.170**	—	—	—	—	—	—	—
6. Ego depletion	43.66	8.07	−0.438**	−0.405**	−0.384**	−0.346**	−0.270**	—	—	—	—	—	—
7. Cognition	17.52	2.87	−0.341**	−0.308**	−0.329**	−0.251**	−0.183**	0.714**	—	—	—	—	—
8. Emotion	13.13	3.18	−0.452**	−0.420**	−0.396**	−0.367**	−0.262**	0.890**	0.531**	—	—	—	—
9. Behavior	13.01	3.80	−0.294**	−0.276**	−0.236**	−0.238**	−0.218**	0.841**	0.319**	0.653**	—	—	—
10. Positive affect	22.61	7.07	0.455**	0.398**	0.452**	0.424**	0.127**	−0.442**	−0.476**	−0.417**	−0.231**	—	—
11. Negative affect	18.55	4.91	0.140**	0.134**	0.195**	0.092	−0.067	0.270**	0.036	0.251**	0.337*	0.210**	—

SD, standard deviation; ^*^p < 0.05; ^**^p < 0.01.

### Path model

First, we used confirmatory factor analysis to test whether the fitting index of our model conformed to the criteria. After adding six paths to modify the model, the results showed that the fit indexes were chi-square test (χ^2^) = 813.234, degree of freedom (df) = 263, χ^2^/df = 3.09 < 4, root mean square error of approximation (RMSEA) = 0.074 < 0.08, comparative fit index (CFI) = 0.894, Tucker-Lewis index (TLI) = 0.879, CFI/TLI = 1.02 > 0.9, and standardized root mean square residual (SRMR) = 0.088. The fit indexes were in the acceptable range, indicating that the model had a good fitting effect.

The path coefficients of the mediation model are shown in Table 3. Self-management was positively associated with positive affect (β = 0.218, *p* = 0.001) and negative affect (β = 0.287, *p* < 0.001) and negatively associated with ego depletion (β = −0.445, *p* < 0.001). Ego depletion was negatively associated with positive affect (β = −0.558, *p* < 0.001) and positively associated with negative affect (β = 0.430, *p* < 0.001).

**Table 3:** Path coefficients of the mediation model (*n* = 383).

**Paths**	**Std.β**	**β**	**SE**	***t***	***p***	**95% CI**
Positive affect → ego depletion	−0.558	−1.469	0.059	−9.519	< 0.001	[−0.679, −0.450]
Negative affect → ego depletion	0.430	1.322	0.070	6.148	< 0.001	[0.292, 0.563]
Positive affect → self-management	0.218	2.500	0.067	3.242	0.001	[0.083, 0.341]
Negative affect → self-management	0.287	3.847	0.058	4.966	< 0.001	[0.174, 0.409]
Ego depletion → self-management	−0.445	−1.942	0.064	−6.921	< 0.001	[−0.585, −0.330]

Std.β, standardized estimate; SE, standard error.

### Mediation analysis

**Figure 1:**
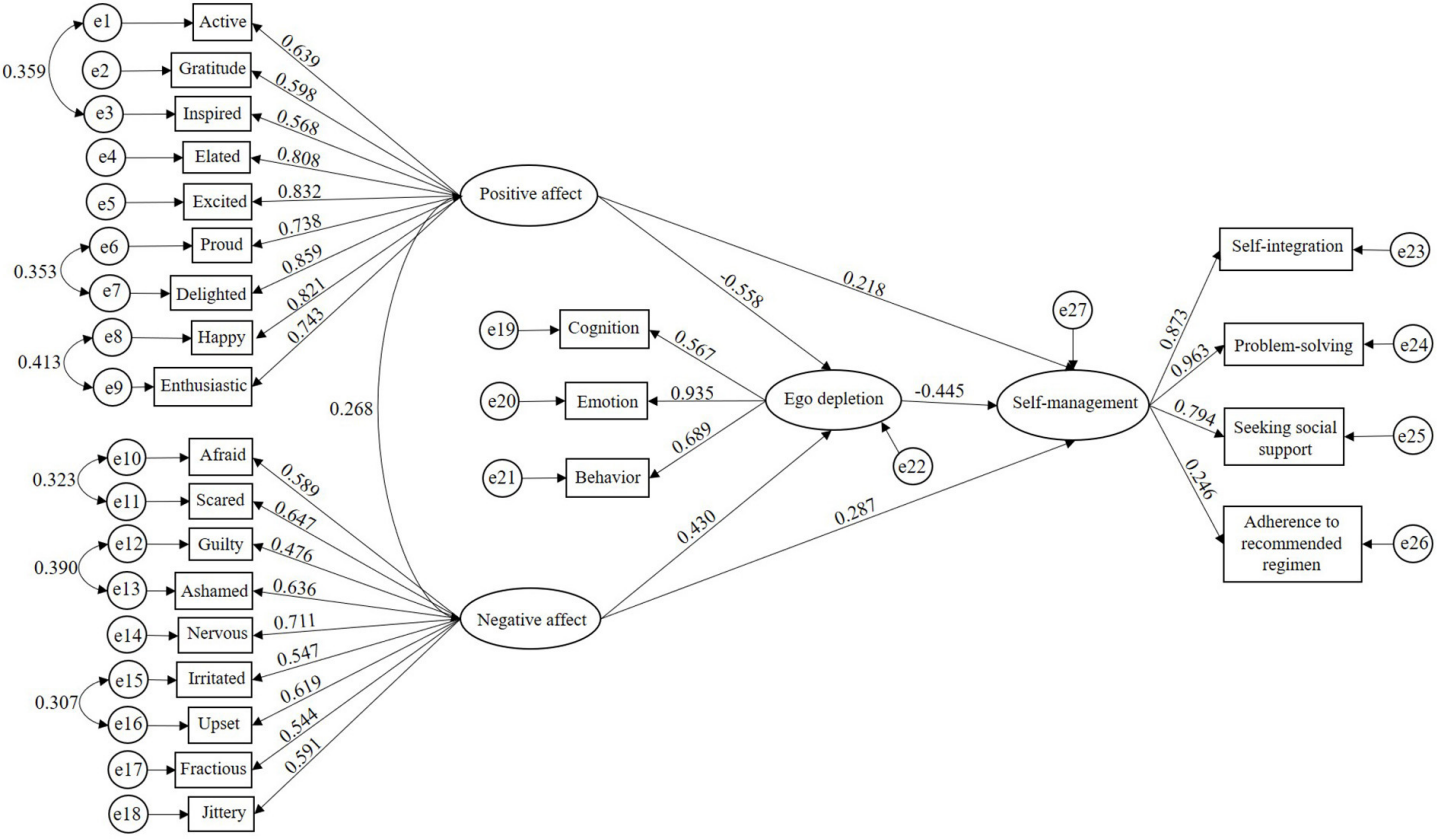
Structural equation model of the mediating effect of ego depletion on the relationships between positive and negative affect and self-management among patients with early CKD (*n* = 383). All factor loadings were significant at the *p* < 0.05 level.

The mediating effect model was tested by 1,000 bootstrapping resamples. The 95% confidence interval of each path is shown in Table 4, and the structural equation model is depicted in Figure 1. The results revealed that the total, direct, and indirect effects of positive affect on self-management through ego depletion were statistically significant. That is, ego depletion played a partial mediating role in the relationship between positive affect and self-management (indirect effect = 0.248, 95% CI: 0.170 to 0.376, *p* < 0.001). The mediating effect of positive affect on self-management accounted for 53.33% of the total effect. However, the direct and indirect effects of negative affect on self-management through ego depletion were statistically significant, while the total effect was not significant. Direct and indirect effects had opposite signs. That is, ego depletion had a suppressing effect rather than a mediating effect on the relationship between negative affect and self-management (indirect effect = −0.191, 95% CI = −0.310 to −0.118, *p* < 0.001). The absolute value of the ratio of the suppressing effect to the direct effect was 66.55%.

**Table 4:** Mediation analysis of ego depletion on the relationship between positive and negative affect and self-management (*n* = 383).

**Paths**	**Effects**	**Std.β**	**β**	***SE***	***p***	**95% CI**
Positive affect → self-management	Total effect	0.466	5.353	0.778	< 0.001	[0.353, 0.617]
	Direct effect	0.218	2.500	0.067	< 0.001	[0.083, 0.341]
Positive affect → ego depletion → self-management	Indirect effect	0.248	2.853	0.634	< 0.001	[0.170, 0.376]
Negative affect → self-management	Total effect	0.096	1.280	0.801	0.110	[−0.032, 0.191]
	Direct effect	0.287	3.847	0.058	< 0.001	[0.174, 0.409]
Negative affect → ego depletion → self-management	Indirect effect	−0.191	−2.507	0.723	< 0.001	[−0.310, −0.118]

Total effect = direct effect + indirect effect; Std.β, standardized estimate; SE, standard error.

## Discussion

Overall, our study validated the mediating effect of ego depletion between positive affect and self-management and discovered the suppressing effect of ego depletion between negative affect and self-management among patients with early CKD, which provided an important theoretical basis and intervention target for improving self-management among them during the COVID-19 pandemic. To the best of our knowledge, this is the first time that the theory of ego depletion has been applied to patients with early CKD, and we paid more attention to these patients during the special period of COVID-19 in China.

Self-management among patients with early CKD needs to be improved. In this study, nearly 60% of participants' self-management was at low and moderate levels, which was consistent with previous studies (36). The symptoms of CKD in early stages are not obvious, and the incidence is more hidden and has little impact on the daily lives of patients, so patients tend to ignore self-management of the disease (2). During the investigation period, due to the pandemic lockdown, some patients could not go to the hospital in time, and there was a lack of guidance for disease prevention and nutrition. Most studies have focused mainly on the nutritional management, dialysis management, and self-management of patients with end-stage renal disease rather than early-stage disease (37, 38). Therefore, it is urgent to pay attention to the self-management of patients with early CKD.

The study also found that patients who were younger, had a higher education level, earned a higher monthly income, had a higher frequency of review, had a lower number of hospitalizations, lived in urban areas, were self-paying for treatment, and had a disease course of < 1 year had better self-management. Younger patients have greater hope for the future and are more eager to control the disease, so they pay more attention to self-management. Patients with higher education levels and higher monthly incomes who live in urban areas may have better knowledge, living conditions and medical services, so they are aware of the importance of self-management during the early stage (39). The economic burden of self-paying patients is heavier than that of patients with medical insurance. To reduce the economic burden, patients work harder to control the disease process and may strengthen self-management. Patients with a disease course of < 1 year are still in the early stage. At this time, strengthening self-management can effectively curb the development of the end stage under the objective fact that the disease cannot be completely cured. The higher the frequency of review and the lower the number of hospitalizations were, the more concern about the development of the disease process and the higher the awareness of the importance of self-management. As a result, from the current situation of self-management among patients with early CKD, we still need to increase the health science knowledge about early CKD. We should make full use of the internet to keep in touch with patients, especially in the face of COVID-19, to improve patients' understanding of the disease, arouse their attention in the early stage, and strengthen self-management to delay progression of the disease.

Ego depletion plays a mediating role in the relationship between positive affect and self-management. That is, positive affect not only had a direct positive predictive effect on self-management but also used ego depletion as a mediating variable to indirectly predict self-management, which was in line with the results of previous studies. Positive affect can help people achieve better self-control (40). Zhu (41) believes that positive affect can help patients perform better in medical decision-making, problem solving and other tasks and can also supplement psychological resources and alleviate ego depletion. The lower ego depletion of a patient can positively predict his or her level of health behavior (42). Patients who usually face life with positive affect such as gratitude, enthusiasm, and inspiration will treat diseases with a more optimistic attitude; they are willing to communicate with family and friends to seek social support and are good at psychological self-regulation. In addition, positive affect can also help patients with early CKD fight against ego depletion by establishing psychological resources through supplementary mechanisms, and finally, their self-management will be improved (43). This also enlightens us that as medical workers, it is very important to mobilize patients' positive affect, help them supplement psychological resources and reduce ego depletion in the usual psychological care, especially during the COVID-19 pandemic. We can apply positive psychological intervention, mindfulness therapy, gratitude therapy, acceptance and commitment therapy to the nursing care of patients with early CKD to help them improve self-management to the greatest extent.

Ego depletion plays a suppressing role in the relationship between negative affect and self-management. The suppressing effect is a kind of mediating effect in a broad sense. The difference between the suppressing effect and the mediating effect is that after controlling for the mediating variable, the effect of the independent variable on the dependent variable decreases; while the suppressing effect is the opposite, after controlling for the suppressing effect, the effect of the independent variable on the dependent variable increases (44, 45). In this study, negative affect was positively related to self-management and ego depletion, while ego depletion was negatively related to self-management. The suppressing effect clearly explained why the total effect of negative affect on self-management had no effect; that is, the direct effect was suppressed by the suppressing variable. This is somewhat different from the point of view put forward by previous studies. For example, Kang (46) found that negative affect such as depression can directly lead to lower self-management. To explore the reasons, first, the different results may be related to the different research times, backgrounds, and populations, especially the different types and degrees of negative affect. Second, we believe that moderate negative affect can indeed stimulate patients' awareness of self-management (17). Negative affect such as guilt and fear will make patients more willing to treat their disease to reduce the family burden and improve their quality of life, so patients will strengthen their self-management. However, as a result of negative affect being positively related to ego depletion, when negative affect continues to accumulate to a certain degree, negative affect will create a negative effect on the human body and consume psychological resources, which will lead to an increase in ego depletion (47). Ego depletion will lead to negative results such as alcoholism, overeating and impulsive anger, which result in the decline of self-management (48, 49). At this point, ego depletion suppresses the stimulating effect of negative affect on self-management. Therefore, it also inspires us that proper control of negative affect and timely supplementation of psychological resources can improve patients' self-management, which also provides ideas for our future qualitative or empirical research.

However, there are some limitations in our study. First, the investigative methods of this study were all patient self-reports, and the results lacked objective indicators, which may lead to bias. In the next study, some objective indicators should be measured to increase the credibility of the results. Second, only three tertiary hospitals in China were selected for this study. In the next step, we should expand the range of sample sizes. Third, the correlation analysis in this study showed that negative affect was positively correlated with self-management, which is contrary to some previous studies. Some studies believed that negative affect was negatively related to self-management (16). However, it also precisely proved that ego depletion played a suppressing role in the relationship between negative affect and self-management. More empirical studies and qualitative studies are needed to verify this finding and further explore the specific mechanism of ego depletion in the relationship between negative affect and self-management in the future. For example, we can consider studying the extent to which negative affect accumulates, resulting in ego depletion, which can provide a more quantitative basis for interventions. It is also possible to clarify the cause of ego depletion through experimental research or qualitative research and provide a scientific basis for intervention measures to improve self-management behavior.

## Conclusion

In conclusion, ego depletion theory and the mediating model could be applied to patients with early CKD during the COVID-19 pandemic. This study explored the mechanism of positive and negative affect on self-management and found that ego depletion played different mediating roles. Positive affect can positively predict self-management and can also improve self-management by reducing ego depletion. Moderate negative affect can positively predict self-management, but when negative affect accumulates to produce ego depletion, ego depletion will play a suppressing role between negative affect and self-management, thus reducing self-management. We have enriched the research on improving self-management among patients with early CKD. To improve self-management, interventions can focus on stimulating patients' positive affect, controlling negative affect, replenishing psychological resources and reducing ego depletion.

## Data availability statement

The raw data supporting the conclusions of this article will be made available by the authors, without undue reservation.

## Ethics statement

The studies involving human participants were reviewed and approved by the Ethics Committee of the Second Affiliated Hospital of Air Force Medical University. The patients/participants provided their written informed consent to participate in this study.

## Author contributions

YC and YZ designed the study. HL was responsible for clinical quality control. RL, HW, and SJ contributed to data collection and analysis. YC and TY drafted the article. NL and YZ critically reviewed the manuscript and made constructive suggestions. All authors contributed to the study and approved the final submission.

## Conflict of interest

The authors declare that the research was conducted in the absence of any commercial or financial relationships that could be construed as a potential conflict of interest.

## Publisher's note

All claims expressed in this article are solely those of the authors and do not necessarily represent those of their affiliated organizations, or those of the publisher, the editors and the reviewers. Any product that may be evaluated in this article, or claim that may be made by its manufacturer, is not guaranteed or endorsed by the publisher.
